# From Value Creation to Sustainability: Exploring the Mediating Effect of Acculturative Isolating Product Advantage in Samarinda’s Iconic Textile Industry

**DOI:** 10.12688/f1000research.178612.2

**Published:** 2026-07-09

**Authors:** Dian Irma Aprianti, Sukisno Selamet Riadi, Herning Indriastuti, Sugeng Hariyadi, Rizky Yudaruddin

**Affiliations:** 1 Management, University Widya Gama Mahakam Samarinda, Samarinda, East Kalimantan, Indonesia; 2 Management, Mulawarman University Faculty of Economy and Business, Samarinda, East Kalimantan, Indonesia; 3 Management, State Polytechnic Samarinda, Samarinda, East Kalimantan, Indonesia

**Keywords:** Acculturative Isolating Product Advantage, Business Sustainability, Creativity, Product Innovation, Value Creation

## Abstract

**Background:**

The sustainability of iconic textile enterprises depends on their ability to transform internal capabilities into enduring competitive advantage. Although value creation, product innovation, and creativity are recognized as key drivers of performance, limited empirical evidence explains how these capabilities translate into long-term sustainability in culturally embedded industries. This study examines the mediating role of acculturative isolating product advantage (AAC) in the Samarinda woven sarong industry, a traditional textile sector characterized by strong cultural identity and craftsmanship heritage.

**Methods:**

A quantitative design using Partial Least Squares Structural Equation Modeling (PLS-SEM) was employed. Data were collected from 109 artisans and business owners in the Samarinda woven sarong industry, East Kalimantan. The measurement and structural models were assessed to test the direct and mediating relationships among value creation, product innovation, creativity, AAC, and business sustainability.

**Results:**

Product innovation and creativity have significant direct effects on business sustainability. Value creation does not directly influence sustainability but exerts a significant indirect effect through AAC. The results confirm that AAC plays a crucial mediating role, indicating that culturally embedded product uniqueness functions as an isolating mechanism that reduces imitation and strengthens long-term viability.

**Conclusions:**

Sustainability in iconic textile enterprises is achieved not merely through generating value, but through transforming that value into culturally distinctive and difficult-to-imitate product advantages. This study extends the Resource-Based View within culturally embedded contexts and clarifies the pathway from value creation to sustainability.

**Recommendations:**

Artisans should strategically manage creativity and innovation to reinforce culturally distinctive products, while policymakers should support innovation capacity, intergenerational knowledge transfer, and culturally based branding strategies. Future studies may adopt longitudinal and comparative approaches to further validate acculturative isolating mechanisms in traditional industries.

## Introduction

The rapid transformation of the global business environment has intensified the emphasis on sustainability, driven by climate change, regulatory pressures, technological disruption, and evolving societal expectations. Contemporary firms are increasingly evaluated not solely on financial performance but also on their ability to ensure long-term viability through responsible governance, adaptive capability, and strategic resource management. In this context, ^1^ argue for the redesign of business models anchored in societal purpose and planetary boundaries, while ^2^ highlight systems thinking as a necessary paradigm shift to transform interconnected technologies, practices, and institutional structures that sustain unsustainable pathways. Within this broader sustainability discourse, value creation, creativity, and innovation are widely recognized as fundamental drivers of resilience, competitiveness, and organizational renewal. However, for iconic textile enterprises rooted in cultural heritage, sustaining relevance in modern markets requires more than technological upgrading or incremental innovation. It demands the strategic transformation of value creation processes into sustainable outcomes through culturally embedded product advantages that are inherently local, symbolic, and socially constructed.

The Samarinda woven sarong industry, rooted in the historical Bugis-Wajo migration to East Kalimantan, represents a distinctive cultural heritage that embodies a fusion of ethnic artistry, local identity, and traditional craftsmanship. As an iconic textile enterprise, this industry reflects how cultural capital is deeply embedded within production processes and market offerings, where weaving techniques, motifs, and symbolic patterns function not merely as aesthetic attributes but as carriers of collective memory and social identity. Similar to the findings of, ^3^ who show that Thai plant-dyed silk integrates ritual practices and local identity directly into production infrastructures, Samarinda sarongs embed intangible heritage into their craftsmanship practices. In parallel, ^4^ demonstrate how traditional symbolism can be systematically translated into contemporary textile design while preserving historical and regional meanings, indicating that cultural authenticity can evolve without losing its embedded value. Moreover, ^5^ argue that high craftsmanship and design traditions in Italian textiles operate as intellectual capital that legitimizes “Made in Italy” as a quality–cultural marker, suggesting that similarly, the Samarinda woven sarong constitutes localized intellectual and cultural capital that differentiates it from mass-produced textiles. Despite this cultural and symbolic strength, the industry faces structural challenges, including generational discontinuity, limited technological modernization, low productivity growth, and increasing competition from factory-based production; regional data indicate that most artisans are senior weavers relying on manual tools, while youth participation remains relatively low, creating structural imbalances that threaten production continuity, the preservation of authenticity, and the intergenerational transmission of tacit knowledge. Consequently, the sustainability of this iconic textile enterprise extends beyond economic viability and represents a broader socio-cultural challenge concerning how deeply embedded cultural capital can be strategically safeguarded and transformed into a durable source of sustainable competitive advantage.

In this context, a critical strategic issue emerges: how can iconic textile enterprises transform value creation, creativity, and product innovation into sustainable competitive advantage while preserving cultural authenticity? Many artisans continue to operate within short-term production orientations, focusing primarily on immediate sales rather than long-term capability development. Value creation, product innovation, and creativity are often present but not systematically managed as strategic resources capable of generating isolating advantages. This situation reveals a broader scientific problem concerning how culturally embedded enterprises can convert internal capabilities into sustained business sustainability in rapidly evolving and competitive markets.

Prior research grounded in the Resource-Based View emphasizes that firms achieve sustained competitive advantage when they effectively deploy valuable, rare, inimitable, and non-substitutable resources. Complementarily, Cultural Capital Theory explains how cultural assets can be transformed into economic value when embedded within products and practices. Empirical studies have demonstrated that value creation, innovation, and creativity positively influence marketing performance and firm competitiveness. ^6, 7^ Other scholars highlight product uniqueness as a strategic source of differentiation and competitive positioning, ^8, 9^ while ^10^ show that acculturative product advantage mediates the relationship between innovation and marketing outcomes.

Nevertheless, the existing literature predominantly emphasizes direct relationships between innovation-based capabilities and performance outcomes, with limited attention to the isolating mechanisms that protect competitive advantage over time. In particular, the transformation from value creation to sustainability remains theoretically underexplored in culturally embedded industries. Culturally embedded product uniqueness is often treated as a differentiation attribute rather than as a strategic isolating mechanism that prevents imitation and sustains long-term advantage. This creates a theoretical tension between cultural differentiation as symbolic value and sustainable competitive advantage as an enduring economic outcome. While cultural authenticity enhances market appeal, it remains theoretically unclear how such authenticity systematically functions as a mediating mechanism that converts value creation, innovation, and creativity into business sustainability. Furthermore, empirical findings regarding the long-term sustainability impact of culturally rooted innovation remain fragmented and occasionally inconsistent, particularly within traditional industries in emerging economies.

Therefore, a significant research gap exists in developing and empirically validating an integrated framework that positions acculturative isolating product advantage as a mediating strategic mechanism linking value creation, product innovation, and creativity to business sustainability. Addressing this gap is essential not only for advancing theoretical integration between resource-based and cultural perspectives but also for clarifying the pathway from value creation to sustainability in iconic textile enterprises.

Drawing on the Resource-Based View framework, this study investigates the influence of value creation, product innovation, and creativity on business sustainability through the mediating role of acculturative isolating product advantage in the Samarinda woven sarong industry as an iconic textile enterprise. By empirically examining these relationships, this research offers three primary contributions. First, it extends the Resource-Based View by incorporating cultural embeddedness into the analysis of sustainable competitive advantage, demonstrating that culturally rooted resources can function as isolating mechanisms rather than merely symbolic differentiators. Second, it advances the application of Cultural Capital Theory within traditional industry contexts by empirically validating acculturative isolating product advantage as a strategic pathway through which value creation and innovation capabilities are translated into long-term sustainability. Third, it provides novel empirical evidence from a traditional textile industry in an emerging economy, offering a comprehensive and contextually grounded model that clarifies the mediating mechanisms through which value creation, creativity, and product innovation collectively drive sustainable business outcomes. Finally, the findings are expected to contribute theoretically by extending the Resource-Based View and Cultural Capital Theory within traditional cultural industries, while also providing practical insights for artisans, SMEs, and policymakers regarding strategies to strengthen sustainability, cultural preservation, and long-term competitiveness in the traditional textile sector.

## Literature review

The Resource-Based View (RBV), which underpins this study, highlights that using resources that are valuable, rare, unique, and non-substitutable can result in a sustained competitive advantage. ^11^ In the context of the Samarinda sarong industry, this theory explains that value creation, product innovation, and creativity serve as strategic assets that strengthen competitiveness and business sustainability. The supporting perspectives from ^12^ on strategic assets and ^6^ on product strategy capability highlight the importance of a firm’s ability to manage cultural and technological assets to create product differentiation. Meanwhile, the Uniqueness Product Advantage (UPA) concept proposed by ^8^ reinforces the notion that product uniqueness rooted in local cultural values and innovative design constitutes an advantage that is difficult to imitate, thereby functioning as an isolating mechanism for sustaining a competitive position. ^9^ This aligns with recent findings showing that creativity and innovation enhance business continuity in traditional industries ^13^ and that operational and infrastructural excellence mediate customer satisfaction and competitive advantage. ^14^

Moreover, in conjunction with the RBV, this study also draws on Cultural Capital Theory, ^15^ which explains how cultural values can be converted into economic and business advantages. Acculturative products that combine local cultural elements with modern features represent a form of objectified cultural capital, embodied in tangible products such as distinctive fabric motifs or traditional weaving techniques. Consumers possessing embodied cultural capital, which reflects their understanding and appreciation of cultural values, tend to show higher loyalty toward such products. Thus, acculturative products hold not only economic value but also symbolic and cultural significance, strengthening their market appeal. The integration of RBV and Cultural Capital Theory provides a robust conceptual foundation, suggesting that acculturative isolating product advantage can serve as a strategic mechanism for achieving long-term business sustainability through cultural differentiation, continuous innovation, and enhanced consumer loyalty. ^16^ highlighted analogous viewpoints, indicating that cultural and environmental values influence purchasing intentions among Gen Z consumers, and by, ^17^ who revealed that cultural knowledge moderates green consumer behavior, demonstrating how embodied cultural capital influences economic outcomes.

The relationships among value creation, product innovation, creativity, acculturative isolating product advantage, and business sustainability demonstrate a multidimensional interaction that intertwines economic, cultural, and social dimensions. Value creation acts as the foundation for sustaining traditional industries by integrating economic viability with social and cultural preservation. ^18, 19^ In the context of the Samarinda woven sarong industry, value creation transforms local wisdom into competitive strategies that enhance both cultural identity and business adaptability. Studies by ^20, 21^ confirm that sustainable value creation is a multilevel process that not only improves firm performance but also engages multiple stakeholders, ensuring that traditional industries maintain relevance in evolving markets. These findings reinforce that embedding ethical, cultural, and environmental dimensions into strategic decision-making contributes significantly to long-term competitiveness. Empirical evidence from SMEs also demonstrates that value creation is reinforced by eco-innovation and environmental collaboration ^22^ as well as by government eco-regulation and institutional support. ^23^

Product innovation complements value creation by enabling industries to develop new designs, techniques, and eco-friendly materials that align with modern consumer preferences while preserving cultural distinctiveness. Within the Samarinda woven sarong context, innovation bridges traditional craftsmanship with contemporary demands, allowing artisans to target younger and international markets. This mechanism aligns with, ^10, 24^ who demonstrate that acculturative product advantages mediate the link between innovation and marketing performance. Similarly, ^6^ emphasize the role of firms’ isolating capabilities in strengthening new product development performance through the integration of competitive experience and marketing resource flexibility, which aligns with the role of innovation in sustaining culturally embedded industries. Thus, innovation serves as both a strategic and adaptive capability that enhances sustainability and market performance. Prior empirical evidence also reinforces this perspective by demonstrating that digital transformation, ^25^ social media engagement, ^26^ and knowledge-based innovation ^27, 28^ enhance SMEs’ adaptive capacity, particularly during periods of systemic disruption such as the COVID-19 pandemic.

Creativity, as the underlying source of innovation, allows artisans to reinterpret traditional motifs, experiment with novel production methods, and integrate storytelling that reflects both heritage and modern aesthetics. This creative process strengthens differentiation while preserving authenticity, ensuring long-term survival in dynamic markets. According to empirical findings presented by, ^29^ creativity plays a crucial role in promoting novelty in entrepreneurial environments by acting as a critical mechanism that connects positive affect to inventive outcomes. ^7^ further highlight that creativity has a strong and significant relationship with innovation, particularly in low-technology industries like handwoven textiles. Complementing these perspectives, ^30^ reveal that creativity enhances consumers’ need for uniqueness, fostering distinctiveness that is crucial for culturally rooted products such as Samarinda woven sarongs. This resonates with empirical evidence by, ^31^ who found that digital adaptation and creative resilience significantly affected small enterprise performance during the crisis, and by, ^32^ who observed that e-commerce utilization boosted business creativity and market reach for small firms.

The concept of acculturative isolating product advantage provides a theoretical link connecting value creation, innovation, and creativity to sustainable business performance. According to, ^9^ isolating mechanisms act as barriers that prevent imitation and secure long-term competitive advantage. Within the Samarinda woven sarong industry, the integration of cultural elements such as motifs, symbolism, and traditional weaving techniques creates authentic differentiation that enhances both the tangible and intangible value of the product. Empirical evidence from ^33^ supports this argument, showing that culturally embedded product characteristics significantly reinforce marketing performance by positioning cultural uniqueness as a central source of advantage. Further contributions from ^34^ suggest that the hybridization of cultural elements with creative design strengthens aesthetic and symbolic value, thereby improving the sustainability of cultural industries through increased global recognition. In addition, research by ^35, 36^ indicates that accounting practices, foresight analysis, and human capital development indirectly enhance entrepreneurial growth and cultural business readiness in Indonesia’s emerging capital region. Although earlier research, such as ^37–39^ emphasizes the value of creativity and innovation in creating a competitive edge, empirical data is still conflicting, particularly when it comes to the long-term sustainability impacts of culturally embedded forms of innovation. The need for more research into the mediating function of acculturative isolating product advantage in tying creativity-based capacities to long-term performance in traditional cultural sectors is highlighted by this conflicting evidence.

**Figure 1.**
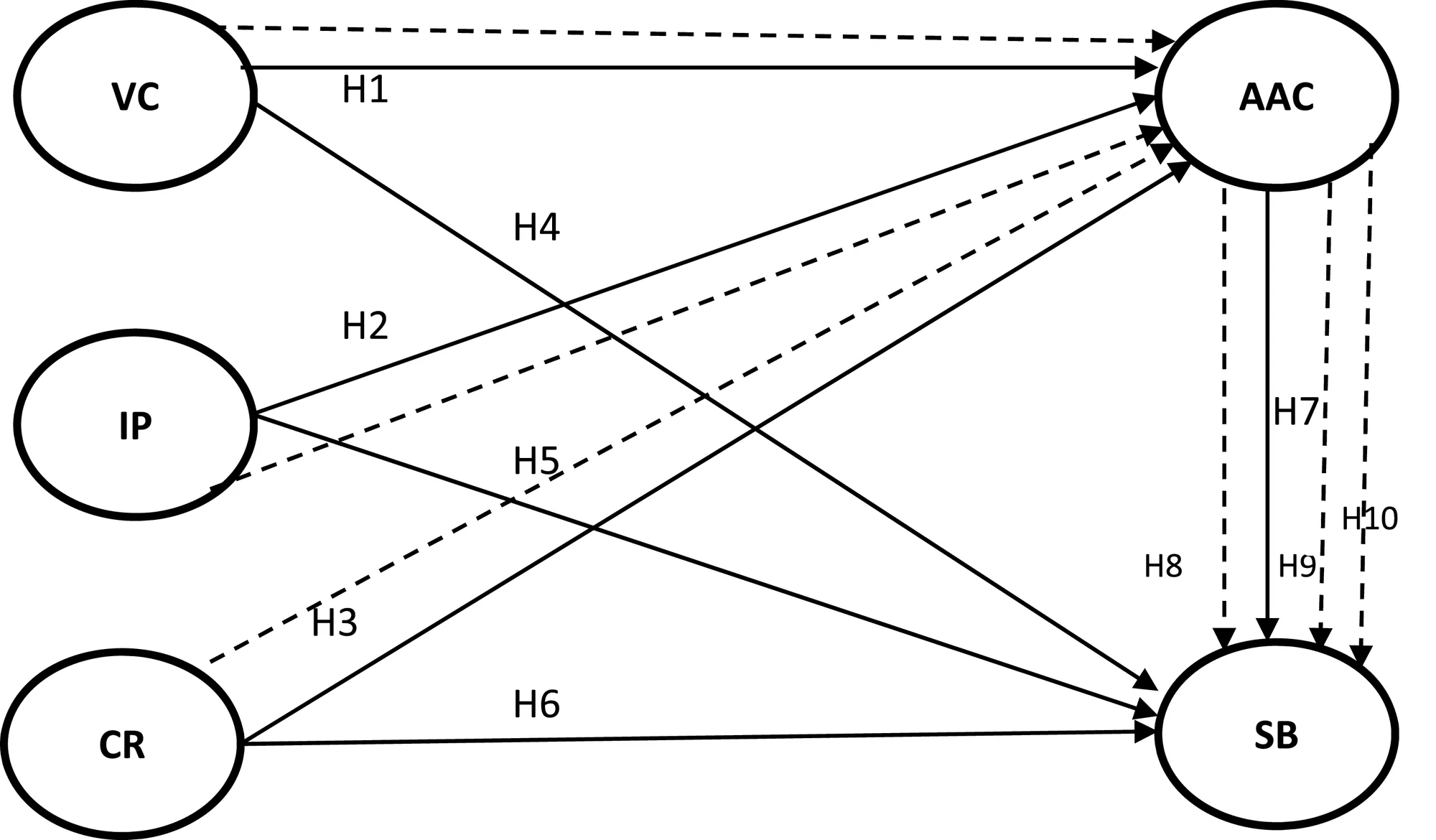
Research framework

Finally, the mediating role of acculturative isolating product advantage reinforces the interaction between internal organizational capabilities (value creation, innovation, creativity) and business sustainability. This perspective aligns with, ^18, 40^ who argue that sustainable strategies must integrate economic profitability with cultural and social responsibility. The Samarinda woven sarong industry exemplifies this balance by leveraging cultural capital as a resource-based capability, ^24^ utilizing innovation as a pathway to market resilience, ^10^ and employing creativity as a source of differentiation. ^7^ In this way, localized cultural capital evolves into a sustainable competitive advantage that preserves traditional heritage while ensuring adaptability and growth within the global marketplace. Recent evidence from ESG and sustainability research also supports this linkage, as shown by, ^41^ where responsible management of ESG risk enhances long-term stability and value creation across firms in emerging markets. This view is consistent with, ^42^ who demonstrate that governance attributes, particularly board structure, significantly influence voluntary environmental and energy disclosure in Indonesian firms, indicating that organizational governance can reinforce sustainability-oriented value creation and long-term business accountability.

In the context of the Samarinda woven sarong industry, acculturative isolating product advantage extends beyond simple product differentiation. Drawing on Rumelt’s isolating mechanism perspective, ^43^ AAC functions as a socially complex and causally ambiguous strategic resource because its cultural authenticity is deeply embedded in tacit weaving knowledge, intergenerational craftsmanship, symbolic meanings, and localized historical identity that cannot be easily replicated by competitors. This argument is consistent with the Resource-Based View, which emphasizes that socially complex and historically embedded resources constitute important sources of sustained competitive advantage. ^11^ Unlike conventional differentiation strategies based solely on design variation, the cultural legitimacy attached to Samarinda woven sarongs emerges from long-standing social recognition and culturally embedded production practices, creating barriers to imitation and sustaining competitive advantage over time. ^32, 33^ From the perspective of Cultural Capital Theory, ^15^ AAC primarily represents objectified cultural capital because cultural values are embodied in tangible product attributes such as motifs, weaving patterns, and symbolic textile identities that simultaneously carry economic and cultural value. Objectified cultural capital is materialized in cultural goods that preserve symbolic meaning while generating social and economic distinction.

Therefore, based on the conceptual model and theoretical framework, the following hypotheses are proposed:

*H1:*
*Value creation has a positive effect on business sustainability.*

*H2:*
*Product innovation has a positive effect on business sustainability.*

*H3:*
*Creativity has a positive effect on business sustainability.*

*H4:*
*Value creation has a positive effect on acculturative isolating product advantage.*

*H5:*
*Product innovation has a positive effect on acculturative isolating product advantage.*

*H6:*
*Creativity has a positive effect on acculturative isolating product advantage.*

*H7:*
*Acculturative isolating product advantage has a positive effect on business sustainability.*

*H8:*
*Value creation mediated by acculturative isolating product advantage has a positive effect on business sustainability.*

*H9:*
*Product innovation mediated by acculturative isolating product advantage has a positive effect on business sustainability.*

*H10:*
*Creativity mediated by acculturative isolating product advantage has a positive effect on business sustainability.*

## Methods

The purpose of this study is to empirically examine the influence of value creation, product innovation, and creativity on business sustainability through acculturative isolating product advantage within the Samarinda woven sarong industry as an iconic textile enterprise of East Kalimantan. As a culturally emblematic industry that represents regional identity and heritage, the Samarinda woven sarong provides a distinctive context for analyzing how culturally embedded capabilities are transformed into sustainable competitive advantage. To achieve this objective, a quantitative research design was employed to provide a structured and measurable analysis of observable phenomena related to sustainable business practices in this iconic traditional textile sector. The research design was developed to test the proposed hypotheses and to generate empirical evidence that supports theoretical confirmation and predictive analysis. The study adopts a confirmatory approach that applies established theoretical frameworks to a specific socio-economic and culturally symbolic context, aiming to identify key factors influencing sustainable competitive advantage in iconic cultural enterprises.

The research population consists of owners and artisans in the Samarinda woven sarong industry, as recorded by the Samarinda Department of Industry, totaling 122 individuals. This study employed a purposive sampling technique by selecting respondents based on specific criteria relevant to the research objectives. The criteria required that participants employ more than two workers, have operated their business for at least five years, and generate an annual turnover exceeding five million rupiah. These criteria were applied to ensure that respondents possessed sufficient business experience and operational stability to evaluate business sustainability practices. Based on these criteria, the final sample comprised 109 active artisans and business owners. These inclusion criteria were intentionally applied to focus on established woven sarong enterprises with relatively stable operational and managerial experience, as such enterprises are considered more capable of evaluating long-term sustainability practices, innovation processes, and culturally embedded competitive strategies. Consequently, newly established micro-businesses and informal household producers that did not meet these criteria were excluded from the analysis. Therefore, the findings of this study should be interpreted within the context of relatively established traditional textile enterprises rather than the broader informal craft sector. Structured questionnaires using a five-point Likert scale, ranging from “strongly disagree” to “strongly agree,” were used to collect primary data. ^44^ The research was conducted in Samarinda, East Kalimantan, chosen for its central role as the economic and cultural hub of traditional weaving industries. Data collection took place from January 2025, covering instrument preparation, empirical data gathering, data analysis, and report completion. The questionnaires were administered directly to respondents and complemented by brief interviews to provide a more comprehensive understanding of business sustainability dynamics in the Samarinda woven sarong industry.

This study classifies research variables into two groups, namely exogenous (independent) and endogenous (dependent) variables. The exogenous variables consist of value creation, product innovation, and creativity. Value creation (VC) refers to the capability of Samarinda woven sarong enterprises to sustain long-term operations by balancing economic, social, and environmental aspects while maintaining acculturative values. It highlights the capacity to create, provide, and seize value for all parties involved, including staff, clients, investors, and the environment. Drawing on, ^45^ four indicators are used to measure value creation: customer added value, differentiation, cost efficiency, and revenue enhancement. The second exogenous variable, product innovation (IP), represents the process of developing new product formats that enhance environmental and social welfare while contributing to economic growth within the woven sarong industry. Based on, ^46^ product innovation is measured by product variety, product design, and product quality. The third exogenous variable, creativity (CR), refers to the transformation of critical evaluation into innovative solutions for products or services that improve value creation. Following, ^47, 48^ creativity is measured using three indicators: curiosity, optimism, and problem detection.

The first endogenous variable, acculturative isolating product advantage (ACC), reflects the ability of acculturative products to serve as cultural icons that represent a specific community, thereby strengthening marketing performance and competitive differentiation. In the context of Samarinda woven sarongs, this advantage arises from the uniqueness of local motifs, styles, and symbols that are difficult to imitate. ^49^ The three indicators for this variable are acculturative motif uniqueness, acculturative design style, and acculturative symbolic identity. The second endogenous variable, business sustainability (SB), refers to the ability of enterprises to maintain operations over the long term through continuous innovation, employee and customer management, and effective resource utilization. According to, ^50^ business sustainability is assessed using three indicators: economic profit, employee welfare, and environmental responsibility. These indicators collectively represent the multidimensional construct of sustainability, integrating financial, social, and ecological performance within the traditional craft industry framework.

PLS-SEM, a technique intended for research with relatively small sample sizes and suitable for analyzing complex interactions among several dimensions, was used to analyze the data in this study. In order to test the research hypotheses and the strength of the relationships among variables, the analysis was conducted in two stages, first evaluating the measurement model to confirm construct validity and indicator reliability, and then evaluating the structural model. ^51^ The path coefficients and their significance levels were estimated using the bootstrapping technique with 5,000 resamples. The model’s explanatory and predictive power were evaluated using coefficient of determination (R ^2^) and predictive relevance (Q ^2^) values. Effect sizes (f ^2^) were also examined to identify the relative influence of each exogenous variable on the endogenous constructs. Hypotheses testing was based on t-values and p-values, with relationships considered statistically significant when p < 0.05 and t > 1.96. This analytical approach ensures the robustness of model estimation and provides clear empirical insights into how value creation, product innovation, and creativity contribute to acculturative isolating product advantage and, subsequently, enhance business sustainability within the Samarinda woven sarong industry.

## Results

The study’s participants comprised 109 entrepreneurs working in the Samarinda woven sarong industry ( Table 1). Most respondents had operated their businesses for more than ten years (81.65 percent), indicating that the majority possess extensive experience in managing and sustaining their enterprises. In terms of workforce size, all respondents (100 percent) reported having more than two artisans involved in production, reflecting the labor-intensive nature of the industry. Regarding average monthly turnover, more than half of the respondents (53.21 percent) earned between IDR 5,000,000 and IDR 10,000,000, while 30.28 percent generated less than IDR 5,000,000, and only 16.51 percent achieved more than IDR 10,000,000. Furthermore, 82.57 percent of respondents started their businesses independently, while 17.43 percent continued family-owned enterprises. These characteristics highlight the dominance of experienced, self-initiated entrepreneurs who play a crucial role in preserving and innovating within the traditional sarong industry in Samarinda.

**Table 1.** Respondent profile.

Criteria	Category	Frequency	Percentage (%)
Business Duration	5–10 years	20	18.35
	> 10 years	89	81.65
Number of Artisans	< 2	0	0
	> 2	109	100
Average Monthly Turnover	< 5,000,000	33	30.28
	5,000,000 – 10,000,000	58	53.21
	> 10,000,000	18	16.51
Business Origin	Started independently	90	82.57
	Family inheritance	19	17.43

The findings of the reliability and construct validity tests for each research variable are shown in Table 2. All items exhibit sufficient indicator reliability and accurately reflect their related constructs, as indicated by the outer loading values for each indicator exceeding the 0.70 threshold. Internal consistency dependability is confirmed by composite reliability (CR) values ranging from 0.925 to 0.957 and Cronbach’s alpha values ranging from 0.910 to 0.949, all of which are higher than the suggested minimum value of 0.70. All constructs have excellent convergent validity, which means that each one accounts for more than half of the variance of its indicators, as indicated by Average Variance Extracted (AVE) values above 0.50. These results indicate that the measurement model meets the criteria for validity and reliability, ensuring that the constructs of value creation, product innovation, creativity, acculturative isolating product advantage, and business sustainability are measured consistently and accurately.

**Table 2.** Construct validity and reliability.

Variable	Indicator	Mean	Standard Deviation	Loadings	Composite reliability	Cronbach’s alpha	AVE
Value Creation (VC)	1. Customer added value (VC1)	4.266	0.789	0.712	0.925	0.910	0.507
	2. Differentiation (VC2)	3.651	0.946	0.728			
	3. Cost efficiency (VC3)	3.972	0.726	0.791			
	4. Revenue enhancement (VC4)	3.881	0.754	0.743			
Product Innovation (IP)	1. Product variety (IP1)	3.890	0.774	0.758	0.936	0.922	0.619
	2. Product design (IP2)	4.101	0.732	0.811			
	3. Product quality (IP3)	4.092	0.714	0.713			
Creativity (CR)	1. Curiosity (CR1)	4.11	0.737	0.852	0.957	0.949	0.712
	2. Optimism (CR2)	3.917	0.883	0.856			
	3. Problem detection (CR3)	3.945	0.792	0.832			
Acculturative Isolating Product Advantage (ACC)	1. Acculturative motif uniqueness (ACC1)	4.275	0.780	0.826	0.938	0.924	0.633
	2. Acculturative design style (ACC2)	4.092	0.866	0.883			
	3. Acculturative symbolic identity (ACC3)	4.037	0.769	0.833			
Business Sustainability (SB)	1. Economic profit (SB1)	4.110	0.774	0.795	0.956	0.947	0.732
	2. Employee welfare (SB2)	4.147	0.650	0.918			
	3. Environmental responsibility (SB3)	4.083	0.709	0.757			

Furthermore, Table 3 demonstrates that the discriminant validity requirements are also satisfied based on the Fornell-Larcker Criterion, where the square root of AVE for each construct exceeds its correlations with other constructs. In addition, the HTMT values are below the recommended threshold of 0.90, confirming that each construct is empirically distinct and adequately discriminated from the others.

**Table 3.** Discriminant validity with Fornell-Larcker Criterion and HTMT.

Fornell-Larcker Criterion					
	VC	IP	CR	ACC	SB
**VC**	0.766				
**IP**	0.822	0.787			
**CR**	0.783	0.835	0.840		
**ACC**	0.710	0.743	0.725	0.831	
**SB**	0.752	0.825	0.830	0.835	0.879

Heterotrait-Monotrait Ratio (HTMT)					
	VC	IP	CR	ACC	SB
**VC**					
**IP**	0.909				
**CR**	0.855	0.894			
**ACC**	0.771	0.793	0.771		
**SB**	0.824	0.888	0.888	0.893	

To minimize the potential risk of common method variance (CMV), several procedural remedies were applied during data collection, including assuring respondent anonymity and emphasizing that there were no right or wrong answers. In addition, a full collinearity assessment was conducted following. ^52^ The inner VIF values presented in Table 4 were all below the recommended threshold of 3.3, indicating that common method bias was not a serious concern in this study.

**Table 4.** Results of inner VIF value.

	VC	IP	CR	ACC	SB
**VC**				3.404	2.531
**IP**				4.360	4.634
**CR**				3.658	3.848
**ACC**					2.521
**SB**					

The results presented in Table 5 confirm that the structural model used in this study demonstrates strong explanatory power and meets all model feasibility criteria. The coefficient of determination (R ^2^) for the mediating variable Acculturative Isolating Product Advantage (ACC) is 0.637, indicating that the exogenous variables value creation (VC), product innovation (IP), and creativity (CR) collectively explain 63.7 percent of the variance in ACC. Meanwhile, the R ^2^ value for Business Sustainability (SB) is 0.831, showing that VC, IP, CR, and ACC together account for 83.1 percent of the variation in business sustainability, which is categorized as very strong. The predictive relevance (Q ^2^) values of 0.638 for ACC and 0.831 for SB further demonstrate the model’s high predictive capability and confirm that the structural model effectively captures the relationships among the studied variables.

**Table 5.** Predictive relevance (Q ^2^) and coefficient of determination (R ^2^).

Construct	R ^2^	R ^2^ Adjusted	Q ^2^
Acculturative Isolating Product Advantage (ACC)	0.64	0.627	0.64
Business Sustainability (SB)	0.83	0.825	0.83

With the exception of Value Creation (VC), which has no discernible direct association with Business Sustainability (SB), the results shown in Table 6 reveal that the majority of independent variables have a significant impact on the dependent variable. Specifically, the path coefficient for VC to SB is −0.053 with a p-value of 0.288 (>0.05), indicating that hypothesis H1 is rejected. In contrast, Product Innovation (IP) and Creativity (CR) have positive and significant effects on SB, confirming that H2 and H3 are accepted. Moreover, VC, IP, and CR each show significant positive effects on Acculturative Isolating Product Advantage (AAC), supporting H4, H5, and H6. Finally, AAC demonstrates a strong and significant influence on SB, leading to the acceptance of H7. Overall, six out of ten hypotheses in the direct-effect model are accepted, highlighting AAC as an important factor linking innovation, creativity, and value creation to business sustainability. Among the significant predictors, AAC shows the strongest direct effect on business sustainability, indicating that culturally embedded competitive advantages play a more central role in sustaining traditional textile enterprises than operational value creation alone. The insignificant direct relationship between VC and SB further suggests that value creation contributes to sustainability only when it is transformed into culturally distinctive and difficult-to-imitate product advantages.

**Table 6.** Direct hypothesis testing.

Hypothesis		Path coefficient	t-Statistic	P-Value	Hypothesis testing	*f* ^*2*^ Effect size
H1	VC → SB	−0.053	0.564	0.288	Rejected	0.221
H2	IP → SB	0.416	4.837	<0.001	Accepted	0.200
H3	CR → SB	0.182	2.000	0.025	Accepted	0.217
H4	VC → AAC	0.293	3.292	<0.001	Accepted	0.042
H5	IP → AAC	0.265	2.978	0.002	Accepted	0.357
H6	CR → AAC	0.286	3.213	<0.001	Accepted	0.152
H7	AAC → SB	0.428	4.977	<0.001	Accepted	0.365

The results shown in Table 7 further confirm the mediating role of Acculturative Isolating Product Advantage (AAC). The path from VC to SB through AAC (VC → AAC → SB) is significant (p = 0.029 < 0.05), while the direct path from VC to SB is not, indicating full mediation. This means that Value Creation (VC) influences Business Sustainability (SB) only through AAC. In contrast, the paths IP → AAC → SB and CR → AAC → SB both demonstrate partial mediation, as Product Innovation (IP) and Creativity (CR) retain significant direct and indirect effects on SB. These findings support H8, H9, and H10, emphasizing the strategic role of AAC as a mediating mechanism connecting Value Creation, Product Innovation, and Creativity with Business Sustainability. These findings indicate that sustainability in culturally embedded textile industries is not driven solely by operational efficiency or innovation capability, but by the ability to convert these internal capabilities into culturally legitimate and socially recognized product advantages.

**Table 7.** Indirect hypothesis testing.

Hypothesis		Path Coefficient	t-Statistic	P-Value	Hypothesis testing	*f* ^2^ Effect size
H8	VC → AAC → SB	0.125	2.18	0.029	Accepted	0.099
H9	IP → AAC → SB	0.113	2.023	0.044	Accepted	0.097
H10	CR → AAC → SB	0.123	2.154	0.032	Accepted	0.102

## Discussion

The results presented in Tables 6 and 7 indicate that Creativity (CR) and Product Innovation (IP) have significant positive effects on Business Sustainability (SB), while Value Creation (VC) shows no direct significant influence. However, VC indirectly influences SB by using Acculturative Isolating Product Advantage (AAC) as a mediator. This pattern highlights that business sustainability in the Samarinda woven sarong industry is not solely driven by the direct creation of value but rather by the transformation of that value into culturally distinctive products. These findings align with, ^19, 18^ who suggest that sustainable value creation emerges when economic performance is combined with social and cultural dimensions. Similarly, ^21, 20^ confirm that value creation in traditional industries involves multi-stakeholder collaboration that bridges cultural identity and competitive advantage, consistent with the empirical outcome that VC contributes indirectly to sustainability through AAC.

The significant effect of Product Innovation (IP) on Business Sustainability (SB) reinforces the idea that innovation serves as a vital mechanism for adapting traditional craftsmanship to modern markets. In the Samarinda woven sarong industry, innovation manifests through improvements in product design, variety, and quality, enabling artisans to remain relevant amid evolving consumer preferences. This finding aligns with the findings of, ^10, 24^ who discovered that acculturative product advantages ensure market resilience by mediating the relationship between marketing performance and innovation. Likewise, ^6^ highlighted that innovation supported by marketing flexibility strengthens product differentiation and competitiveness. Hence, the current findings confirm that innovation not only enhances market adaptability but also fosters sustainability by bridging tradition and modernity within the woven sarong sector.

Innovation in traditional textile industries increasingly involves the adoption of sustainability-oriented technologies and adaptive production practices. Recent studies on textile innovation emphasize that emerging technologies in the textile sector are closely associated with environmental sustainability, circular production systems, and resource efficiency. ^53^ In the context of the Samarinda woven sarong industry, technological adaptation does not necessarily imply full industrial automation, but rather selective innovation that preserves cultural authenticity while improving product quality, design flexibility, and market responsiveness. However, similar to the challenges identified by Cardona-Acevedo et al., traditional textile enterprises often face operational constraints such as limited financial resources, low technological capability, generational discontinuity, and resistance to organizational change. These barriers explain why innovation and creativity become critical strategic capabilities for sustaining competitiveness in culturally embedded industries.

Furthermore, the findings may also be interpreted through the perspective of circular economy and sustainable cultural production. Prior studies such as ^54^ argue that circularity in textile industries requires the integration of sustainability, local knowledge, and long-term resource management within sector-specific production systems. In developing regions, traditional textile enterprises frequently encounter greater structural limitations than firms in developed economies, including restricted access to technology, weaker institutional support, and limited innovation infrastructure. Nevertheless, culturally embedded industries such as the Samarinda woven sarong sector possess unique symbolic and social capital that can strengthen sustainable competitiveness through authenticity-based differentiation. Therefore, the sustainability pathway in developing-country textile industries may depend less on large-scale technological transformation and more on the strategic integration of cultural preservation, creativity, and adaptive innovation.

Creativity also demonstrates a significant positive influence on both AAC and SB, confirming its essential role as a source of differentiation and innovation in traditional industries. This finding supports the theoretical framework proposed by, ^29^ who found that creativity mediates positive affect and innovation outcomes, and, ^7^ who emphasized creativity’s importance in generating innovative ideas in low-technology industries such as handwoven textiles. Moreover, ^30^ showed that creativity enhances consumers’ perceptions of uniqueness, which is particularly relevant for culturally embedded products. In the context of Samarinda woven sarongs, creativity empowers artisans to reinterpret cultural symbols and transform them into innovative product forms that appeal to modern consumers without losing authenticity. Thus, creativity serves as the foundation for achieving both differentiation and sustainability.

The mediating effect of Acculturative Isolating Product Advantage (AAC) confirms its strategic function as a cultural and competitive mechanism linking VC, IP, and CR to SB. As noted by, ^9^ isolating mechanisms act as protective barriers that sustain long-term competitive advantage, particularly when rooted in cultural uniqueness. In this study, AAC partially mediates the routes of IP and CR to SB and entirely mediates the relationship between VC and SB, demonstrating how cultural difference improves the conversion of creativity and invention into long-lasting results. This is consistent with the findings of, ^33^ who demonstrated that acculturative design and symbolic identity enhance product value and market longevity. Therefore, AAC functions as a strategic bridge that transforms internal capabilities into sustainable competitive advantage, confirming that business sustainability in traditional industries is most effectively achieved when cultural authenticity is combined with innovation and creativity.

## Conclusion

This study aims to empirically examine the influence of value creation, product innovation, and creativity on business sustainability through the mediating role of acculturative isolating product advantage (AAC) within the Samarinda woven sarong industry. A quantitative method and PLS-SEM analysis were used to gather information from 109 Samarinda, East Kalimantan, business owners and artisans. The findings reveal that product innovation and creativity have significant positive effects on business sustainability, while value creation indirectly influences sustainability through AAC. This result indicates that sustainable competitiveness in traditional industries is not merely built through value creation but through the transformation of such value into culturally distinctive and innovative products. The empirical evidence confirms that AAC serves as a key mediating mechanism linking internal capabilities such as innovation and creativity to long-term sustainability by enhancing cultural differentiation and adaptive innovation.

Policymakers and business professionals can benefit from the study’s practical consequences in a number of ways. Local governments and industry stakeholders should prioritize programs that support innovation, creativity, and cultural product development among artisans to strengthen their sustainable competitiveness. Efforts such as training in creative design, digital marketing, and intellectual property protection can further enhance AAC as a strategic advantage. However, the conclusions of this study may not be as broadly applicable as they may be due to its narrow emphasis on a single business and geographic area. The methodology could be extended to other creative sectors or geographical areas in future studies to examine trends in sustainability and cultural innovation. Additionally, incorporating qualitative approaches may provide deeper insights into how cultural values interact with business strategies in achieving long-term sustainability. However, this study should also be interpreted in light of the rapidly evolving nature of sustainability, digital innovation, and technology adoption within the textile sector. Emerging technologies, changing consumer preferences, and evolving sustainability regulations may influence the long-term applicability of the current findings, particularly in traditional industries facing adaptive and technological pressures. Future research is therefore encouraged to examine how digital technologies, AI-assisted design, sustainability-oriented production systems, and circular economy practices can be integrated into culturally embedded textile industries. Further studies may also explore the role of government policies, innovation ecosystems, and institutional support in facilitating sustainable competitiveness among traditional textile enterprises in developing economies.

## Ethical approval and consent to participate

This study received ethical approval prior to data collection from the Ethics Committee of the Faculty of Economics and Business, Widya Gama Mahakam University, Indonesia, in collaboration with the Faculty of Economics and Business, Mulawarman University (Ethical Approval No. 17/2025). The Ethics Committee serves as the institutional review board responsible for evaluating research involving human participants. All research procedures were conducted in accordance with the ethical standards established by the reviewing institutions and relevant institutional guidelines. Written informed consent was obtained from all participants prior to their participation in the study. Participation was voluntary, and strict confidentiality and anonymity of respondents were maintained throughout the research process.

## Consent to publish declaration

We, the authors, agree to publish this work in F1000Research and confirm that the manuscript is original, has not been previously published, and is not under consideration for publication elsewhere. We also affirm that any personal data included in this study have been used with the explicit consent of the individuals concerned, and documented evidence of such consent is available upon request.

## Data Availability

Underlying and extended data: Yudaruddin, R. (2026). Questionnaire Results [Data set]. Zenodo. https://doi.org/10.5281/zenodo.18859759 This repository contains the fully anonymised respondent-level dataset used in the statistical analyses, together with the survey instrument, participant information sheet, informed consent template, and the variable operationalisation framework. All materials are distributed under a Creative Commons Attribution 4.0 International licence (CC-BY 4.0). The data and supporting documents may be reused in accordance with the terms of this licence.
