# Supplementary material for: From Value Creation to Sustainability: Exploring the Mediating Effect of Acculturative Isolating Product Advantage in Samarinda’s Iconic Textile Industry
Source: F1000Res. 2026 Jul 9;15:494. Originally published 2026 Apr 10. [Version 2] doi: 10.12688/f1000research.178612.2 (PMC13555051; doi:10.12688/f1000research.178612.2)
Supplement: Peer review history

# Reviewer report: Reviewer response for version 2

**DOI:** 10.5256/f1000research.201806.r504881

**Published:** 2026-07-28

**Authors:** Tejendra Singh Gaur, Rishiraj Singh Gaur

**Affiliations:** 1 Manipal University Jaipur, Jaipur, Rajasthan, India

The introduction may be strengthened by more clearly highlighting the research gap and explicitly stating how the study contributes to the existing body of knowledge.

 The literature review could benefit from the inclusion of a few recent studies published within the last two to three years to better reflect the current state of research.

 The methodology section may be elaborated slightly by providing additional justification for the selection of the research method and sample to improve transparency and reproducibility.

 Some tables and figures may be improved by ensuring consistent formatting, clearer captions, and uniform numbering throughout the manuscript.

 The discussion section could include a more detailed comparison of the findings with those reported in previous studies to better position the study within the existing literature.

 The manuscript may be carefully proofread to correct minor grammatical errors, improve sentence flow, and ensure consistency in terminology and formatting

Is the work clearly and accurately presented and does it cite the current literature?

Partly

If applicable, is the statistical analysis and its interpretation appropriate?

Partly

Are all the source data underlying the results available to ensure full reproducibility?

Partly

Is the study design appropriate and is the work technically sound?

Partly

Are the conclusions drawn adequately supported by the results?

No

Are sufficient details of methods and analysis provided to allow replication by others?

Partly

Reviewer Expertise:

Circular economy; sustainability; waste management; Industry 4.0/5.0; waste management

We confirm that we have read this submission and believe that we have an appropriate level of expertise to confirm that it is of an acceptable scientific standard, however we have significant reservations, as outlined above.

# Reviewer report: Reviewer response for version 2

**DOI:** 10.5256/f1000research.201806.r500075

**Published:** 2026-07-13

**Authors:** Jan Muhammad Sohu, Quswah Makhdoom

**Affiliations:** 1 Sukkur IBA University, Sukkur, Pakistan; 2 Business, Shaheed Zulfikar Ali Bhutto Institute of Science and Technology, Hyderabad, Sindh, Pakistan

No further comments to make.

Is the work clearly and accurately presented and does it cite the current literature?

Partly

If applicable, is the statistical analysis and its interpretation appropriate?

Partly

Are all the source data underlying the results available to ensure full reproducibility?

Partly

Is the study design appropriate and is the work technically sound?

Partly

Are the conclusions drawn adequately supported by the results?

Partly

Are sufficient details of methods and analysis provided to allow replication by others?

Partly

Reviewer Expertise:

Value Co-creation, Sustainability Digital Marketing, AI, SMEs, Green Innovation,

We confirm that we have read this submission and believe that we have an appropriate level of expertise to confirm that it is of an acceptable scientific standard.

# Reviewer report: Reviewer response for version 1

**DOI:** 10.5256/f1000research.197021.r476782

**Published:** 2026-05-13

**Authors:** Jan Muhammad Sohu, Quswah Makhdoom

**Affiliations:** 1 Sukkur IBA University, Sukkur, Pakistan; 2 Business, Shaheed Zulfikar Ali Bhutto Institute of Science and Technology, Hyderabad, Sindh, Pakistan

The manuscript investigates the influence of value creation (VC), product innovation (IP), and creativity (CR) on business sustainability (SB), with acculturative isolating product advantage (AAC/ACC) positioned as a mediating variable in the context of the Samarinda woven sarong industry of East Kalimantan, Indonesia. Drawing on the resource-based view (RBV) and cultural capital theory, the authors employ PLS-SEM with data from 109 artisans and business owners. The findings indicate that product innovation and creativity directly influence sustainability, while value creation operates only indirectly through AAC. The study positions cultural embeddedness as an isolating mechanism for sustained competitive advantage in heritage-based industries. While the topic is timely and the contextual contribution is valuable, the manuscript contains numerous structural, statistical, and editorial inconsistencies that must be addressed before it can be considered scientifically sound.

**Detailed Comments:**

**Comment #1:** The literature review proposes ten hypotheses (H1–H10), with H7–H9 specifically formulated as mediation hypotheses (VC/IP/CR → AAC → SB) and H10 as the direct effect of AAC on SB. However, Table 4 labels the direct effect of AAC → SB as "H7" (it should be H10), and Table 5 labels the mediation paths as H8, H9, and H10 (they should be H7, H8, H9). Compounding this, the results narrative states, "Six out of seven hypotheses are accepted," even though ten hypotheses were originally formulated. This is a fundamental error that undermines the internal coherence of the manuscript and must be corrected throughout.

**Comment #2:** The Methods section states that "saturated sampling" was used to include all 122 individuals in the population, yet the final sample was reduced to 109 based on three filtering criteria (more than two workers, at least five years of operation, and annual turnover exceeding five million rupiah). Saturated sampling, by definition, includes the entire population without further filtering. The authors are actually applying purposive or criterion-based sampling. This terminological misapplication needs to be corrected, and the rationale for sample selection should be clearly justified.

**Comment #3:** The mediating variable is defined as "ACC" in the Methods section ("acculturative isolating product advantage (ACC)") but is referred to as "AAC" throughout the Abstract, Results, Discussion, and Tables 4 and 5. Given that this construct is the theoretical heart of the paper, the inconsistency is particularly damaging. A single, consistent abbreviation should be selected and applied uniformly across the manuscript, including the conceptual model, tables, and narrative.

**Comment #4:** In Table 2, the Creativity construct is presented as "Creativity (VC)" with indicators labeled VC1 (Curiosity), VC2 (Optimism), and VC3 (Problem Detection). These should be labeled as CR1, CR2, and CR3 since "VC" is already used to denote value creation elsewhere in the same table. This duplication causes confusion regarding indicator identification and reflects a careless oversight in measurement reporting.

**Comment #5:** The authors cite Hair et al. (2019) as their methodological guideline for PLS-SEM, yet they fail to report any discriminant validity statistics. Neither the Fornell-Larcker criterion nor the HTMT (Heterotrait-Monotrait) ratio of correlations is presented. This is a non-negotiable requirement of a rigorous PLS-SEM measurement model. Without discriminant validity, it is impossible to confirm that the constructs (VC, IP, CR, AAC, SB) are empirically distinct from one another.

**Comment #6:** The Methods section explicitly states, "Effect sizes (f²) were also examined to identify the relative influence of each exogenous variable on the endogenous constructs." " However, no f² values appear anywhere in the Results. Either the analysis was not actually conducted, or the table was omitted. This violates basic standards of methodological transparency and replicability.

**Comment #7:** For PLS-SEM, the assessment of multicollinearity using Variance Inflation Factor (VIF) values is mandatory before structural model evaluation. No VIF values are reported, which leaves the structural estimates vulnerable to bias from inter-construct collinearity, particularly relevant given that VC, IP, and CR are conceptually overlapping antecedents.

**Comment #8:** The Discussion opens with "The results presented in Table 5.4 indicate that…" There is no Table 5.4 in the manuscript. This appears to be a residual reference from an earlier dissertation or chapter-style draft and indicates inadequate proofreading. It should be corrected to "Table 4" (and Table 5 where mediation effects are discussed).

**Comment #9:** A study built around a complex mediation model with four constructs and ten hypotheses must include a visual conceptual framework diagram. None is provided. Readers must reconstruct the model mentally from textual descriptions, which is unacceptable for a PLS-SEM publication. A figure showing all hypothesized paths (H1–H10) with their corresponding arrows should be added before the hypotheses are listed.

**Comment #10:** Because all variables (VC, IP, CR, AAC, SB) were measured from the same respondents using a single survey instrument with Likert scales, the manuscript is highly susceptible to common method variance. The authors do not mention Harman's single-factor test, full collinearity assessment, or any procedural remedy. This is a critical methodological omission for self-report cross-sectional data.

**Comment #11:** The t-statistic for H3 (CR → SB) is reported simply as "2" rather than to two or three decimal places (e.g., 2.000 or 2.245), which is inconsistent with the other entries in the table. Furthermore, with t = 2 the corresponding p-value should be approximately 0.046 (two-tailed), yet 0.025 is reported. Similarly, the p-value of 0.288 for H1 is unusually high relative to a t = 0.564, suggesting possible reporting confusion between one-tailed and two-tailed significance. The threshold rules ("p < 0.05 and t > 1.96") declared in the Methods imply two-tailed testing, but several p-values appear consistent with one-tailed tests. Clarification

 and recalculation are required.

**Comment #12:** Several references contain clear errors: References 22 and 23 list authors as "Achmad GN, et al., et al., et al." (triple "et al."), which is a formatting glitch. Reference 43 ends with "6th Editio" (truncated). Reference 39 (Keller et al.) has a malformed year/title structure ("2003Advantages Strong Brands"). Citation style throughout the text is also awkward ("Drawing on four indicators…"; "According to isolating mechanisms…"), placing the comma before the superscript number, which is non-standard.

**Comment #13:** No descriptive statistics (means, standard deviations, ranges) are provided for any of the five constructs or their indicators. This omission, combined with the lack of a correlation matrix among latent variables, makes it impossible for readers to assess the data distribution or replicate the analysis even with the deposited dataset.

**Comment #14:** The title refers to "Iconic Textile Enterprises" (plural, generic), implying broad applicability. However, the empirical study is confined to a single industry (Samarinda woven sarong) in a single regional context (East Kalimantan), with N = 109. The conclusions about "iconic textile enterprises" are therefore not adequately warranted by the data. Either the title should be narrowed to reflect the specific case, or the limitations section should explicitly acknowledge that generalizations to other iconic textile contexts (Italian fashion, Thai silk, Indian khadi, Japanese kimono) are not empirically supported.

**Comment #15:** The manuscript claims to "extend the Resource-Based View by incorporating cultural embeddedness," but the theoretical mechanism by which cultural authenticity functions as an *isolating mechanism* (à la Rumelt, 1984) is not rigorously developed. The discussion conflates "differentiation" with "isolating mechanism," yet these are distinct theoretical constructs in the RBV literature. Additionally, the integration with cultural capital theory (Bourdieu) is superficial: the distinction between embodied, objectified, and institutionalized cultural capital is mentioned briefly in the literature review but never operationalized in the measurement model. The theoretical contribution should be sharpened by clearly articulating (a) what specific mechanism makes acculturative product features causally ambiguous or socially complex enough to deter imitation, and (b) which form of cultural capital the AAC construct empirically represents.

**Additional Minor Observations**

 The abstract contains the duplicated header "Abstract*" beneath "Abstract," a formatting artifact. The historical claim regarding the "Bugis-Wajo migration to East Kalimantan" in the Introduction lacks a supporting citation. The Limitations section is brief and does not address cross-sectional design constraints, self-report bias, or social desirability bias. The recommendations in the abstract mention longitudinal and comparative approaches, but these are not consistently echoed in the conclusion's future research agenda.

Is the work clearly and accurately presented and does it cite the current literature?

Partly

If applicable, is the statistical analysis and its interpretation appropriate?

Partly

Are all the source data underlying the results available to ensure full reproducibility?

Partly

Is the study design appropriate and is the work technically sound?

Partly

Are the conclusions drawn adequately supported by the results?

Partly

Are sufficient details of methods and analysis provided to allow replication by others?

Partly

Reviewer Expertise:

Value Co-creation, Sustainability Digital Marketing, AI, SMEs, Green Innovation,

We confirm that we have read this submission and believe that we have an appropriate level of expertise to confirm that it is of an acceptable scientific standard, however we have significant reservations, as outlined above.

# Reviewer report: Reviewer response for version 1

**DOI:** 10.5256/f1000research.197021.r476775

**Published:** 2026-05-06

**Authors:** Sebastián Cardona-Acevedo

**Affiliations:** 1 Instituto Tecnológico Metropolitano, Medellín, Colombia

Congratulations on this manuscript. It presents a coherent and relevant contribution by linking AI, sustainability, and circular economy practices within a specific industrial context. The structure is solid, and the integration of technological and environmental perspectives reflects a strong understanding of current research trends. Below are several comments aimed at strengthening the depth and positioning of the paper.

1. The introduction clearly frames the relevance of sustainability and AI, although it could further emphasize the sector-specific dynamics that shape innovation processes. Incorporating insights such as those discussed in *Cardona-Acevedo et al. (2026)* on the textile innovation process would help contextualize how these dynamics operate in practice.
2. The research objectives are well defined, but they could be sharpened by explicitly linking them to measurable outcomes or expected contributions to both theory and practice.
3. The methodology is rigorous and transparent; however, a deeper reflection on inclusion/exclusion criteria would improve clarity regarding the scope of the analysis.
4. The results are well organized, though some sections remain descriptive. Strengthening the analytical narrative would improve the interpretation of patterns and trends.
5. The identification of technological trends is a strong point. This section could be reinforced by aligning findings with studies such as *Cardona-Acevedo et al. (2026)* on emerging technologies for environmental sustainability in the textile industry, highlighting convergence in technological pathways.
6. The discussion provides a good synthesis, but it would benefit from a more explicit connection between technological adoption and operational challenges within industries.
7. The circular economy perspective is well integrated; referencing frameworks like those explored in *Quiroz-Fabra et al. (2025)* would strengthen the theoretical grounding regarding circularity in sector-specific contexts.
8. The geographic distribution of research is insightful, although further reflection on disparities between developed and developing regions would add critical depth.
9. The limitations section is appropriate, yet it could include a discussion on the fast-evolving nature of AI and sustainability research, which may affect the longevity of findings.
10. The figures and tables are clear and useful, but their integration into the discussion could be more explicit to reinforce key arguments.
11. The conclusion summarizes the study effectively, though it would be stronger with a clearer articulation of future research directions, particularly in terms of technological implementation and policy implications.
12. Overall, the manuscript offers a valuable contribution by bridging AI, sustainability, and circular economy, though it would benefit from deeper sectoral contextualization and stronger analytical interpretation.

Is the work clearly and accurately presented and does it cite the current literature?

Partly

If applicable, is the statistical analysis and its interpretation appropriate?

Not applicable

Are all the source data underlying the results available to ensure full reproducibility?

Partly

Is the study design appropriate and is the work technically sound?

Yes

Are the conclusions drawn adequately supported by the results?

Partly

Are sufficient details of methods and analysis provided to allow replication by others?

Partly

Reviewer Expertise:

My experience in the field is associated with technological innovation and sustainability, including circular economy, in the textile sector.

I confirm that I have read this submission and believe that I have an appropriate level of expertise to confirm that it is of an acceptable scientific standard, however I have significant reservations, as outlined above.

## References

[1] : From Value Creation to Sustainability: Exploring the Mediating Effect of Acculturative Isolating Product Advantage in Iconic Textile Enterprises. *F1000Research* .2026;15: 10.12688/f1000research.178612.1 10.12688/f1000research.178612.1 PMC1355505142723669

[2] : The innovation process: analysis of the particularities in the textile sector. *Journal of Innovation and Entrepreneurship* .2026; 10.1186/s13731-026-00646-5 10.1186/s13731-026-00646-5
